# Epidemic Wave Dynamics Attributable to Urban Community Structure: A Theoretical Characterization of Disease Transmission in a Large Network

**DOI:** 10.2196/jmir.3720

**Published:** 2015-07-08

**Authors:** Anne G Hoen, Thomas J Hladish, Rosalind M Eggo, Michael Lenczner, John S Brownstein, Lauren Ancel Meyers

**Affiliations:** ^1^ Computational Epidemiology Group Children's Hospital Informatics Program Boston Children's Hospital Boston, MA United States; ^2^ Department of Pediatrics Harvard Medical School Harvard University Boston, MA United States; ^3^ Department of Epidemiology Geisel School of Medicine at Dartmouth Dartmouth College Hanover, NH United States; ^4^ Emerging Pathogens Institute Department of Biology University of Florida Gainesville, FL United States; ^5^ Department of Integrative Biology The University of Texas at Austin Austin, TX United States; ^6^ Île Sans Fil Montreal, QC Canada; ^7^ Santa Fe Institute Santa Fe, NM United States

**Keywords:** communicable diseases, epidemics, transmission

## Abstract

**Background:**

Multiple waves of transmission during infectious disease epidemics represent a major public health challenge, but the ecological and behavioral drivers of epidemic resurgence are poorly understood. In theory, community structure—aggregation into highly intraconnected and loosely interconnected social groups—within human populations may lead to punctuated outbreaks as diseases progress from one community to the next. However, this explanation has been largely overlooked in favor of temporal shifts in environmental conditions and human behavior and because of the difficulties associated with estimating large-scale contact patterns.

**Objective:**

The aim was to characterize naturally arising patterns of human contact that are capable of producing simulated epidemics with multiple wave structures.

**Methods:**

We used an extensive dataset of proximal physical contacts between users of a public Wi-Fi Internet system to evaluate the epidemiological implications of an empirical urban contact network. We characterized the modularity (community structure) of the network and then estimated epidemic dynamics under a percolation-based model of infectious disease spread on the network. We classified simulated epidemics as multiwave using a novel metric and we identified network structures that were critical to the network’s ability to produce multiwave epidemics.

**Results:**

We identified robust community structure in a large, empirical urban contact network from which multiwave epidemics may emerge naturally. This pattern was fueled by a special kind of insularity in which locally popular individuals were not the ones forging contacts with more distant social groups.

**Conclusions:**

Our results suggest that ordinary contact patterns can produce multiwave epidemics at the scale of a single urban area without the temporal shifts that are usually assumed to be responsible. Understanding the role of community structure in epidemic dynamics allows officials to anticipate epidemic resurgence without having to forecast future changes in hosts, pathogens, or the environment.

## Introduction

Epidemics of infectious diseases are frequently characterized by multiple waves of infection [1-3]. Notably, the 1918 influenza pandemic spread through several US and European cities in multiple waves with local variation in the frequency and timing of individual epidemic peaks [4-8]. Predicting when and where disease will resurge is critical to effective prevention and control. However, the drivers and dynamics of multiwave epidemics are unclear. For influenza pandemics, possible explanations include antigenic drift [8-12], waning immunity [13], changing environmental conditions [12,14,15], and social distancing behavior [15-17].

Community structure—aggregation into highly intraconnected but loosely interconnected groups—is a common feature of social contact networks [18] that can potentially drive multiwave epidemics as a disease spreads through one group before emerging in another. However, community structure has been neglected as a possible explanation for multiwave influenza pandemics, in part because it is difficult to detect and estimate [19]. Most studies describing routine human contact patterns have relied on diary- or questionnaire-based surveys [20] or specially deployed wireless sensors [21] and, thus, rarely yield data sufficient for inferring large-scale aggregations. Social networks estimated from electronic “contacts” (ie, cell phones, social networking websites) have been shown to exhibit community structure at larger scales [22-26], but do not capture the physical interactions through which diseases spread. However, the ubiquity of community structure across these networks suggests that it may be a general hallmark of social networks.

Here, we address the hypothesis that contact patterns in a large, empirical, urban contact network are sufficient to generate multiwave epidemics for pandemic influenza-like diseases in the absence of any temporal changes in the hosts, pathogen, or environment. We find that the fate of an epidemic in such a network—whether and when multiple waves occur—depends not only on community structure but also, critically, the presence or absence of bridge superspreaders who forge connections between communities. Direct links between the popular members of different communities synchronize outbreaks; the occasional absence of such bridges provides the epidemiological separation underlying multiwave epidemics.

Interactions between strangers can serve as critical transmission routes for respiratory diseases such as influenza, yet they are difficult to capture in traditional sociological surveys. Using data indicating the physical proximity of more than 100,000 Wi-Fi hotspots users, we characterize the structure of an urban extrasocial interaction network and assess its epidemiological implications.

##  Methods

### Data

Île Sans Fil (ÎSF) is a not-for-profit organization established in 2004 in Montreal, Canada, that operates a system of public Internet hotspots. Hotspots are located in cafes, community and recreation centers, salons, markets, and other small businesses and public places. They are maintained by ÎSF staff and volunteers with the Internet connection provided by the establishment. We analyzed the database of all connections to the system of 352 hotspots between August 2004 and March 2010. Raw data from the ÎSF database consisted of 2.18 million connection records. Each record included an anonymized user ID, latitude and longitude coordinates for each ÎSF hotspot location, connection and disconnection times, and the unique media access control address for the user’s wireless device. The data reported in this paper are available from the Community Resource for Archiving Wireless Data at Dartmouth (CRAWDAD) archive [27].

### Network Construction

We built a contact network by interpreting each individual user as a node and concurrent ÎSF usage at the same hotspot as an edge. This preliminary network contained 114,810 nodes and 1.2 million edges. It contained both self-loops (users connecting multiple devices at once) and parallel edges (pairs of users with multiple overlapping hotspot visits) that we removed to produce a nonredundant network with 637,430 edges. We analyzed the largest connected component of this network, which consisted of 103,425 nodes and 630,893 edges.

### Community Structure Analysis

Modularity (Q) quantifies the extent of community structure in a network relative to a comparable random network. Given a network and a particular partitioning of the nodes into communities, Q is defined as the number of edges contained within communities minus the number of edges expected to fall within communities if the edges were distributed randomly (preserving the degrees of all nodes), normalized for network size. Q ranges from zero for randomly connected networks to greater than 0.3 for networks with substantial community structure [28]. We used a heuristic method to divide the Montreal network into a set of communities that maximized Q using an algorithm [28] that initially assigned each node to its own community and then iteratively aggregated whichever pair of communities resulted in the largest increase in Q. We identified 1420 distinct communities associated with a Q value of 0.69.

### Epidemic Simulations

Epidemic curves were simulated by EpiFire [29] using a chain-binomial [30] network epidemic simulator with a susceptible-infectious-recovered state progression. Each epidemic begins with all nodes susceptible except for a single, randomly selected, infectious node. Nodes remain infectious for a fixed period with arbitrary time units, after which they become recovered for the remainder of the simulation. Transmission from infectious nodes to susceptible neighbors is attempted once per time unit with transmission probability T_cb_. We assumed an infectious period of 5 time units because it yielded sufficient temporal resolution to distinguish epidemic waves. T_cb_ relates to the percolation transmission probability as given by T=1–(1–T_cb_)^d^, where *d* is the infectious period. We simulated epidemics across 3 transmission scenarios defined by the value of the basic reproduction number, R_0_, which is defined as the expected number of secondary cases produced by a typical infection in a completely susceptible population: a low R_0_=1.9, similar to estimates for recent influenza pandemics [31-33]; a moderate R_0_=2.4, which maximizes the probability of multiwave epidemics and is in the range estimated for the 1918 influenza pandemic in the United States [34,35]; as well as a high R_0_=7.5, where spread is rapid both within and between communities. Finally, we performed a sensitivity analysis with respect to the length of the infectious period and considered values of 1, 5, 10, and 50 time steps.

### Detecting Multiwave Epidemics

To automatically identify epidemic curves exhibiting multiple waves, we defined a new 2-peak metric (*TP*), indicating the depth of the deepest valley in the epidemic time series (specifically, the geometric mean of the heights of on each side of the deepest valley). Additional details are provided in Multimedia Appendix 1. Example epidemic curves and their corresponding value of *TP* are shown in the supplementary information (see Figure S1 in Multimedia Appendix 2). The distribution of *TP* values tended to be bimodal with single-wave epidemics yielding values close to zero and multiwave epidemics yielding higher values (see Figure S2 in Multimedia Appendix 2).

### Percolation-Based Approximations of R_0_, Epidemic Size, and Community Bridging

We adapted methods from percolation theory [36] to estimate global and community-specific values of R_0_, final epidemic sizes, and epidemiological connectivity among different communities to test the hypothesis that multiwave epidemics occur in the absence of between-community degree assortativity. The details and derivations of these percolation-based quantities can be found in Multimedia Appendix 1.

### Network Shuffling

To examine the epidemiological impact of community structure we constructed “null” networks that shared many properties of the original Montreal network (eg, the degree distribution) but lacked community structure. Specifically, we iteratively randomized the network by shuffling connections; that is, we chose random pairs of connections and swapped the ends (eg, A-B, C-D became A-D, C-B). This slowly degraded community structure while preserving the number of contacts for each individual. We selected a fraction *f* of edges and broke them to form stubs (half-edges still attached to their nodes). Then, the list of stubs was randomized and each sequential pair of stubs was connected. We eliminated newly formed self-edges and redundant edges via edge swaps with randomly chosen edges, leading to randomization of slightly more than the intended fraction of edges (~1.01*f*). Modeling each community as a semirandom network with the observed within- and between-community degree distributions, we estimated the impact of random shuffling and variation in R_0_ on the epidemiological proximity of communities. We built 2048 new networks by randomly shuffling from 0.1% of edges (631 edges) to 3% of edges (18,927 edges) in increments of 0.1%. All estimates are averages based on the networks built for each shuffling level.

## Results

**Figure 1:**
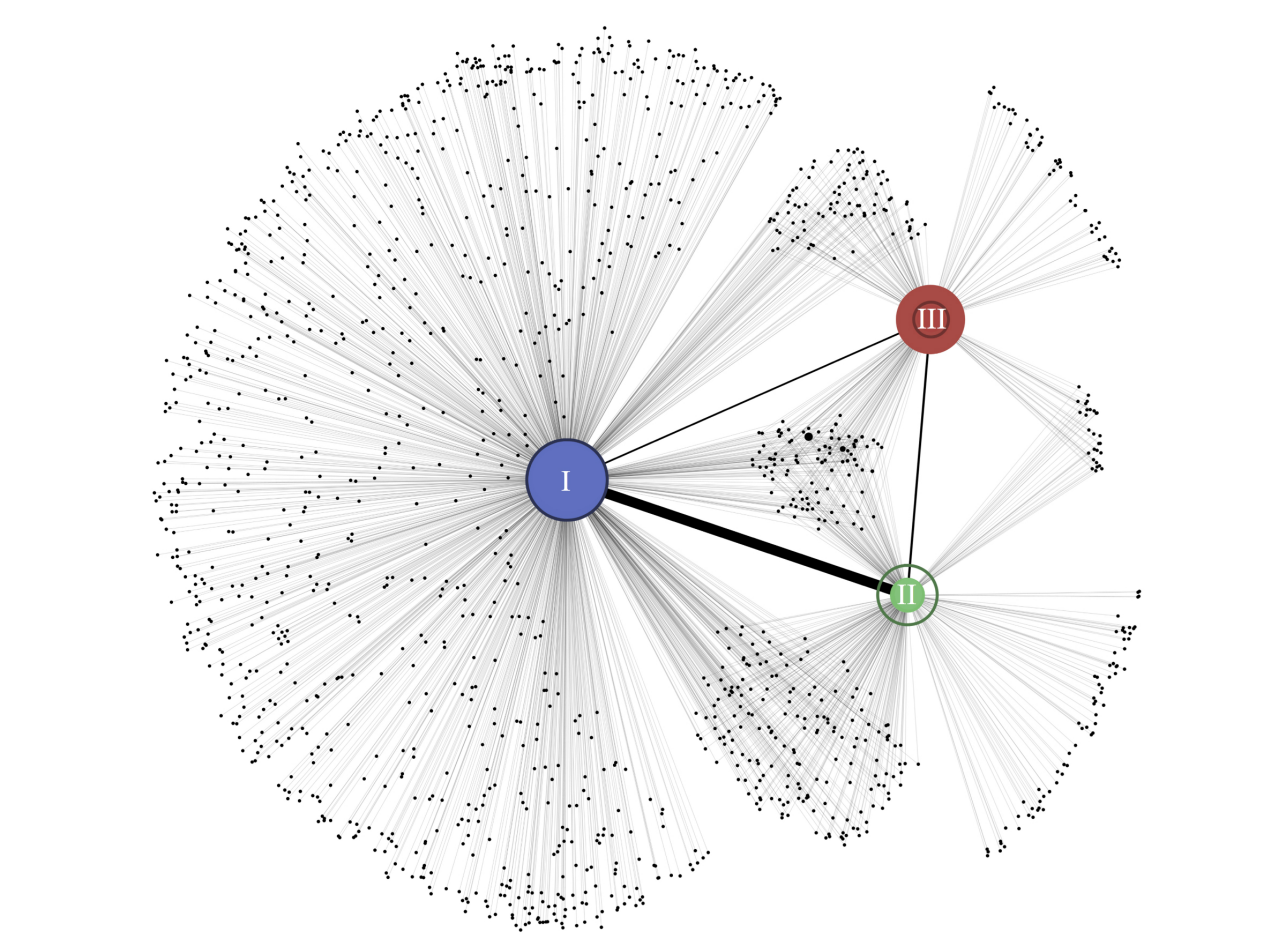
Visualization of connectivity between 1420 communities identified at maximum modularity (Q). Each circle represents a community with filled circle diameter indicating the relative number of within-module edges. Lines joining pairs of communities are drawn with a thickness that is proportional to the number of edges connecting them. The largest 3 communities (community I, II, and III) are labeled and filled in color. Darker rings superimposed on communities I, II, and III are proportional in diameter to the number of nodes making up each.

**Figure 2:**
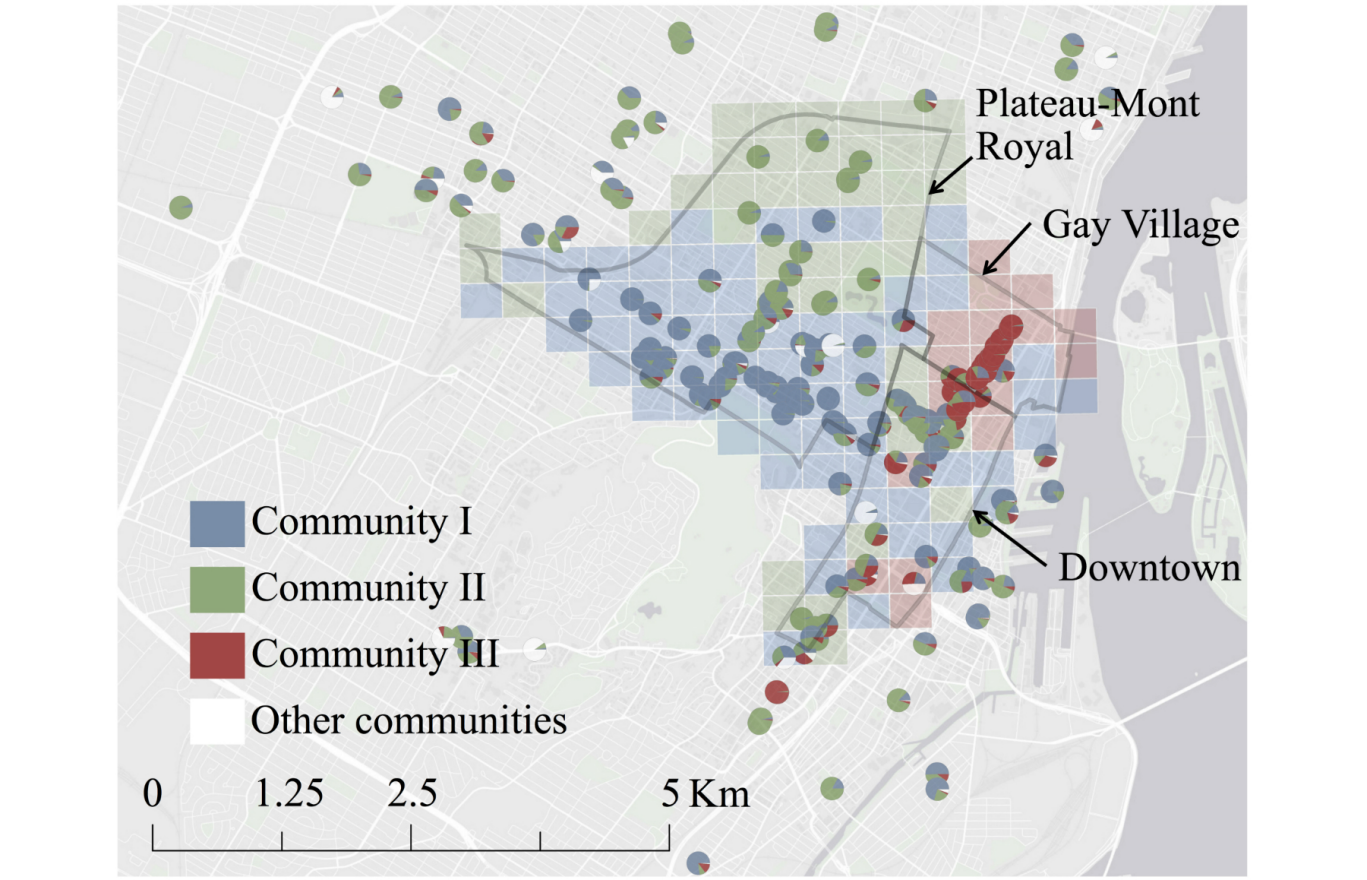
Map of hotspot locations with points on map represented by pie charts indicating the relative contributions of each community to the total visits recorded at that hotspot. Community III was primarily concentrated in the Gay Village neighborhood of the Ville-Marie borough of Montreal, whereas communities I and II primarily occupied the high-traffic commercial areas on either side of the Plateau-Mont-Royal neighborhood; all 3 communities coincided downtown. Each grid square is colored to represent the locally dominant community. Squares with no hotspots are colored to represent the dominant community at the nearest hotspot.

We analyzed the network of more than 600,000 physically proximal contacts between 103,425 users of a free public Wi-Fi hotspot system in Montreal, Canada (hereafter the Montreal network) to examine the effects of ordinary urban contact patterns on epidemic wave dynamics. We used an established heuristic method [28] to divide the Montreal network into 1420 distinct communities (Figure 1; also see Figure S3 in Multimedia Appendix 2). The 3 largest communities together contained 82,228 of 103,425 (79.50%) users in the network, with 38,569 (community I), 28,101 (community II), and 15,558 (community III) users. The mean degree (number of contacts) per user in each of these communities was 13.4 (SD 40.2), 8.3 (SD 23.7), and 26.7 (SD 76.4), respectively, compared with a mean of 4.6 (SD 9.3) for the 21,197 (20.50%) remaining users. The communities exhibited distinct geographic signatures corresponding to large mixed commercial and residential areas in the city, with considerable overlap occurring in Downtown Montreal (Figure 2).

**Figure 3:**
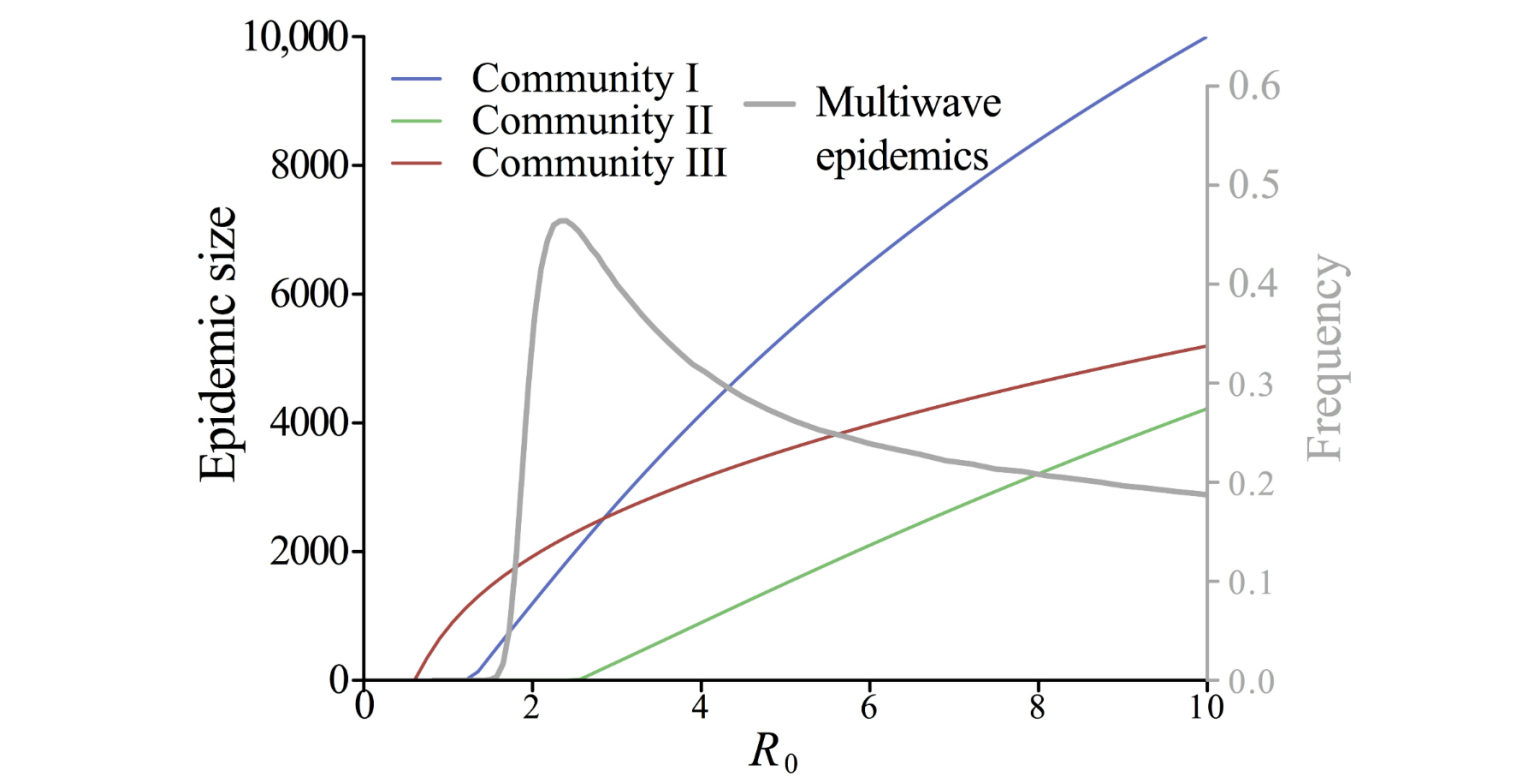
Expected within-community epidemic size assuming that communities were approximately random networks and maintained their empirical within-community degree distributions (colored lines; primary y-axis). The epidemic threshold for each community (ie, R_0_ value for which transmission is sustained) is lowest for community III, followed by communities I and II. The frequency of multiwave epidemics depended on R_0_ and is highest when R_0_=2.4 (gray line; secondary y-axis).

R_0_ is related to the likelihood and extent of a sustained outbreak [37]. R_0_ depends on the transmissibility of the pathogen, host recovery, and the structure of the host contact network [38]. Assuming that within-community contacts are approximately random and using a percolation-based model [39], we estimated that a disease with a global R_0_ equal to 1 had local R_0_ values of 0.8, 0.4, and 1.6 in communities I, II, and III, respectively (when considering only within-community edges) and exhibited considerable variability in epidemiological vulnerability across communities (Figure 3).

**Figure 4:**
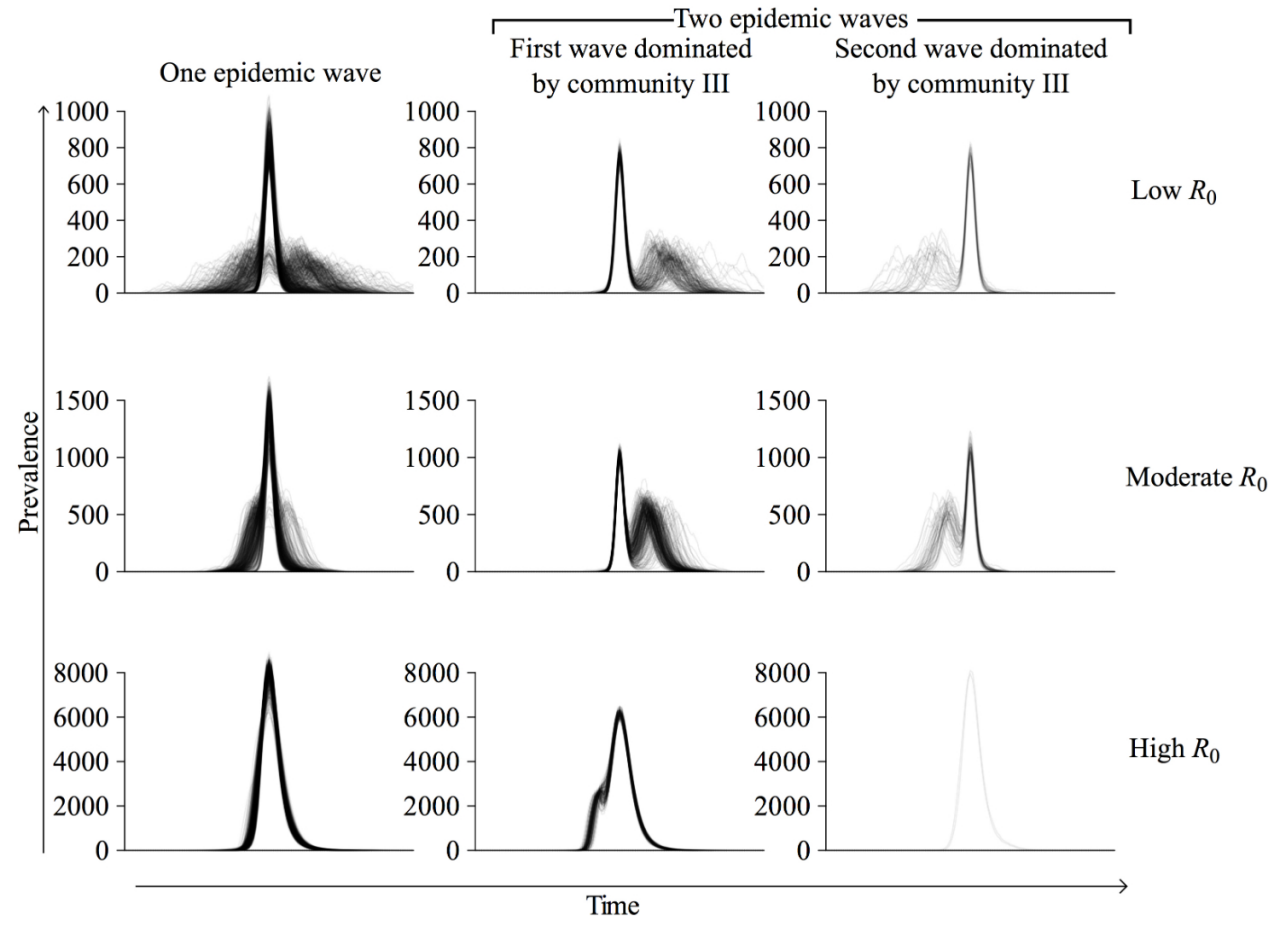
Taxonomy of epidemic curves. For each R_0_, 1000 simulated epidemics were classified as either single-wave (left), multiwave starting with a community III wave followed by a community I and II wave (middle), or multiwave ending in a community III wave (right). At R_0_=2.4, 554 of 1000 (55.40%) had a single wave and 446 of 1000 (44.60%) had 2 waves; of the 446 with 2 waves, community III dominated the first wave in 392 (87.89%). At R_0_=1.9 and R_0_=7.5, only 22.20% (222/1000) and 20.60% (206/1000) of epidemics exhibited 2 waves, respectively. Time series are superimposed so that the peaks of the largest waves align.

**Figure 5:**
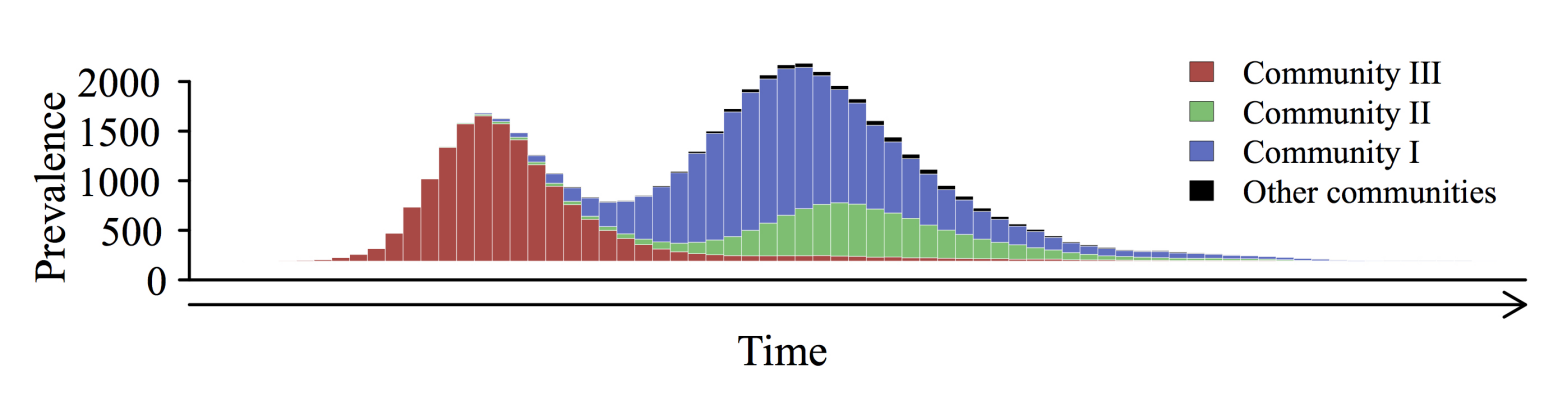
A typical epidemic curve with 2 waves (R_0_=3.7). Community III drove the first wave; communities I and II drove the second wave.

We simulated epidemics through the Montreal network with a stochastic susceptible-infected-recovered model [37] across a range of R_0_ values. At low R_0_, only 2 of 3 communities (I and III) sustained transmission, whereas at high R_0_, epidemic spread was relatively synchronized between communities. Under both of these scenarios, multiwave epidemics were possible but relatively infrequent (Figure 3). At an intermediate value, in the range estimated for the 1918 influenza pandemic in the United States (R_0_=2.4) [34,35], 44.60% (446/1000) of all epidemics exhibited multiple waves and 87.9% (392/446) of these had an initial epidemic wave in community III with a subsequent wave dominated by the 2 larger communities (Figures 4 and 5). When the first wave was dominated by community III, its peak occurred a mean of 27 time steps (SD 7; n=4074) before that of the second wave. When the second wave was dominated by community III, its peak lagged behind the peak of the first wave by a mean of 22 time steps (SD 8; n=572). The relative size of the second wave increased with R_0_ because community III became epidemiologically saturated more quickly than the other 2 communities (Figures 3 and 4). Sensitivity analysis suggested that these results are robust to the length of the infectious period (see Figure S4 in Multimedia Appendix 2).

**Figure 6:**
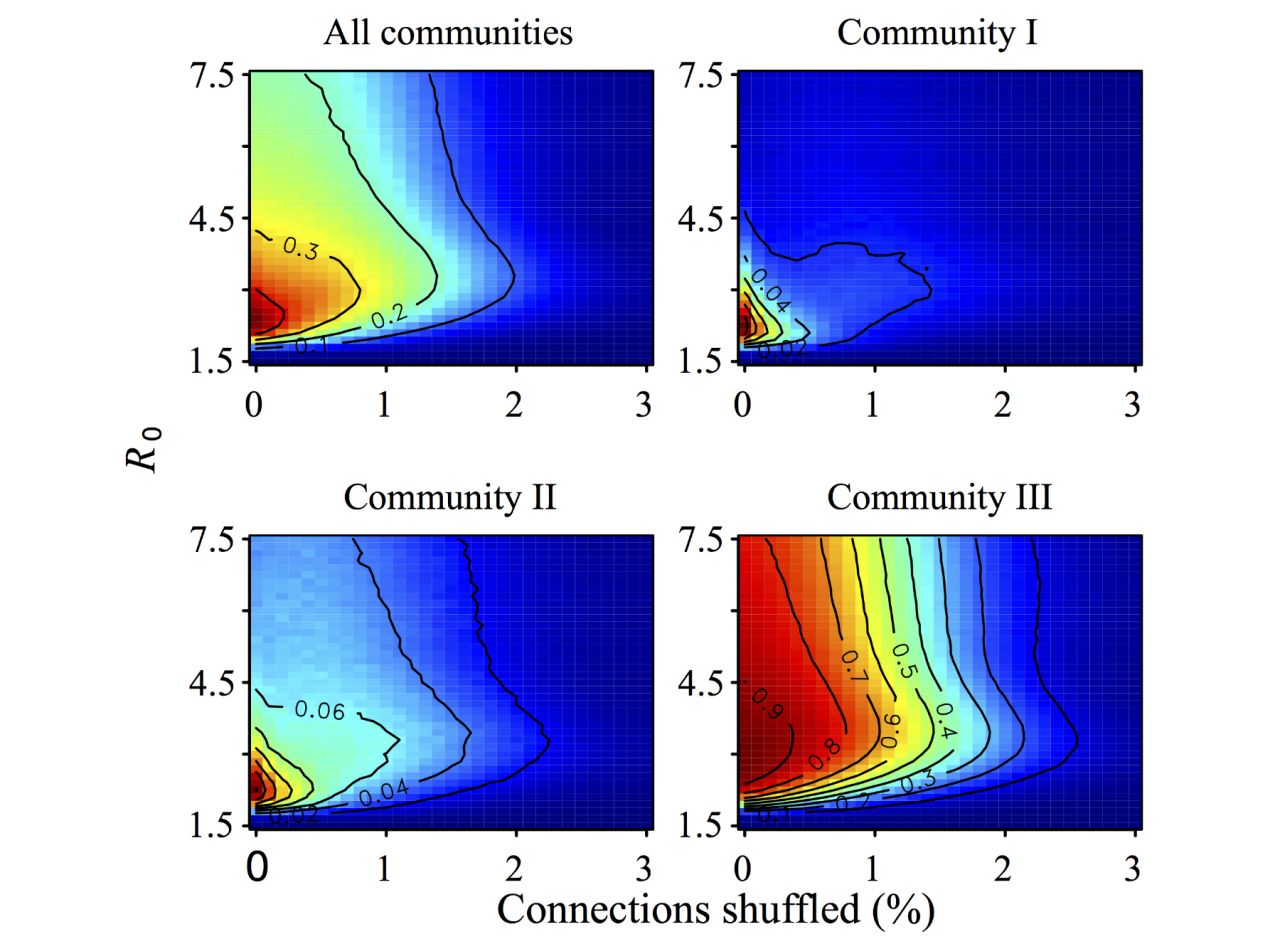
The frequency of multiwave epidemics at varying values of R_0_ and network shuffling with warm colors indicating a higher proportion of multiwave epidemics and cool colors indicating a low proportion of multiwave epidemics (frequency values indicated on contours). Frequencies were calculated across all epidemics (top left) and stratified by starting community (top right and bottom). Each pixel is based on 81,920 simulated epidemics originating in the specified community (10 simulations on each of 8192 uniquely shuffled networks).

**Figure 7:**
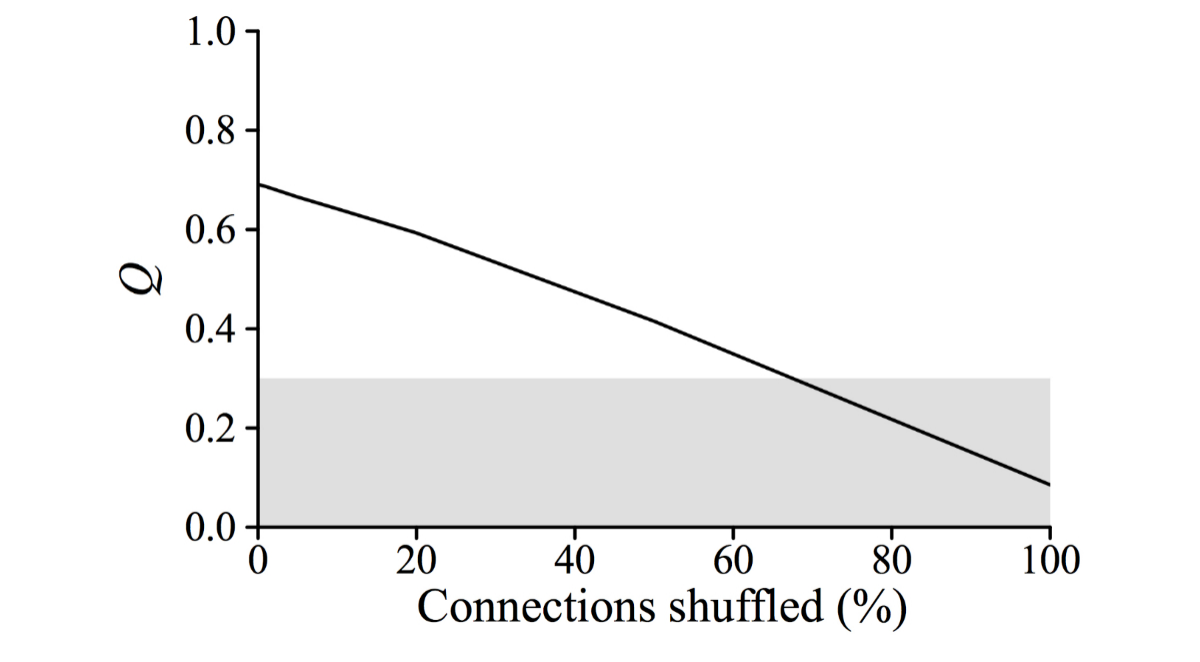
Relationship between modularity (Q) and network shuffling. Shaded area indicates the conventional community structure threshold of 0.3. Note larger x-axis range in this figure compared with other figures.

A modest amount of network shuffling (<3% of edges) almost entirely eliminated multiwave epidemics (Figure 6), whereas it minimally impacted the extent of community structure according to standard metrics including Q (Figure 7) and the numbers of edges linking distinct communities (see Figure S5 in Multimedia Appendix 2). This suggests that the critical driver of 2-peaked epidemics is not the number but the nature of intermodule contacts. We hypothesized that epidemiological synchrony arises when locally popular users in one community tended to be connected to locally popular users in another (between-community degree assortativity*)* and multiwave epidemics can occur only when communities lack such connectivity.

**Figure 8:**
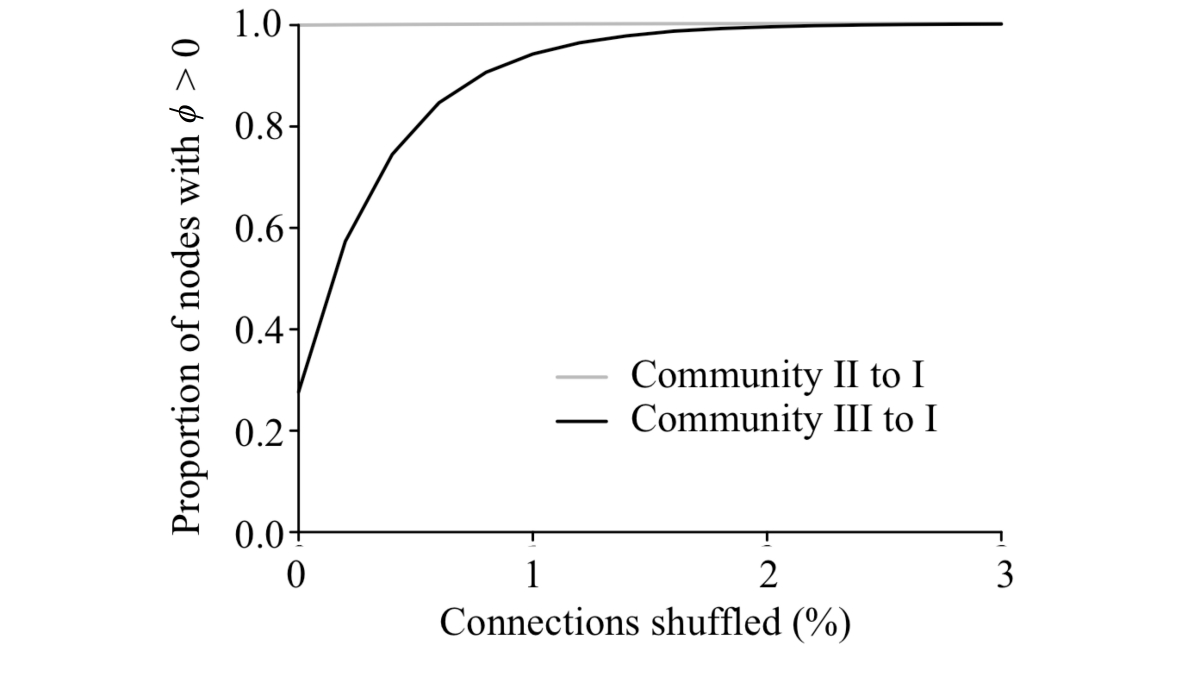
The proportion of nodes in community II (gray line) and community III (black line) with the ability to spark an epidemic (i.e., ϕ >0; see Multimedia Appendix 1) in module I across varying levels of network shuffling at R_0_=2.4.

**Figure 9:**
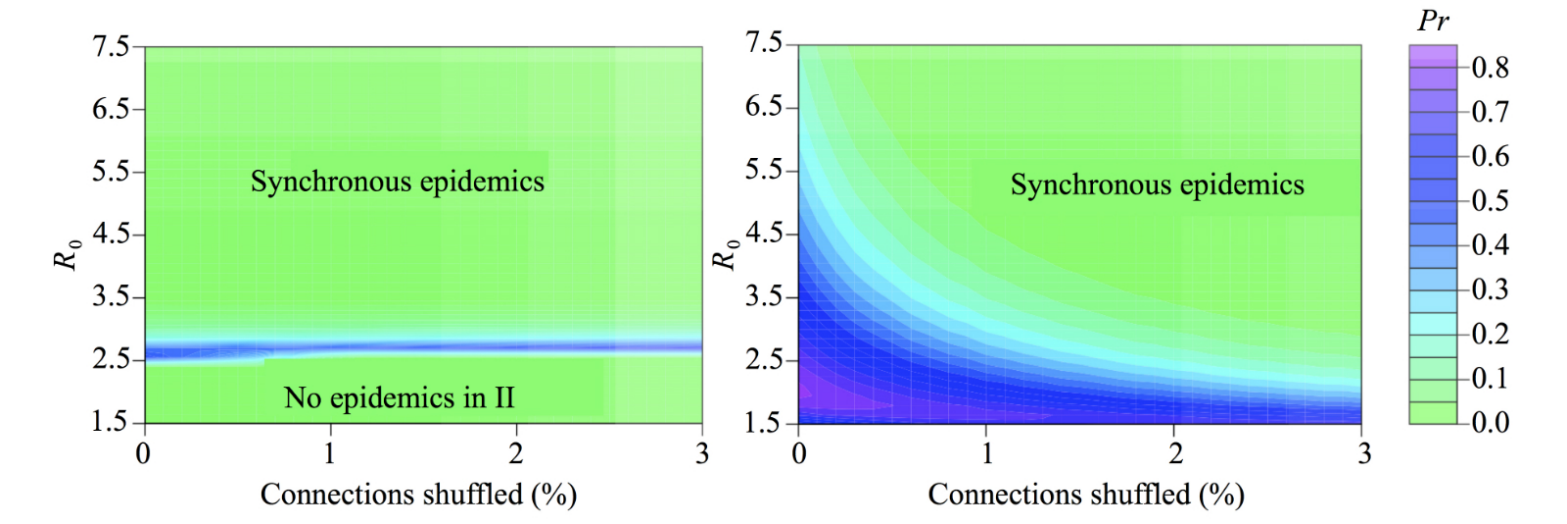
The probability that one community will seed an epidemic in another sufficiently late to produce an asynchronous secondary wave is shown for epidemics spreading from community I to II (left) and community III to I (right).

We formalized and tested this idea by assuming, again, that within-community edges form semirandom networks and used new percolation-based estimates to characterize the epidemiological bridges between the 3 major communities. Given the degree distribution of the Montreal network (see Figure S6 in Multimedia Appendix 2), we found that the number of users in community III expected to form epidemiological bridges to community I increased rapidly with shuffling, whereas communities I and II were tightly connected by bridging individuals in the original network and this connection persisted through shuffling (Figure 8). Shuffling also led to a rapid decrease in the probability that an epidemic starting in community III would spark an epidemic in community I sufficiently late to appear 2-peaked (Figure 9). The precipitous decline in two-wave epidemics with shuffling coincides with the rapid creation of epidemiological bridges and decrease in the expected waiting time between community outbreaks.

The rapid deisolation of the internally well-connected community III occurred because random shuffling targeted users proportional to their degree, which tended to connect high-degree individuals inside community III with high-degree users from elsewhere in the full network. The locally popular but highly insular users of community III thereby quickly formed bridges to popular users in the other major communities.

## Discussion

### Principal Findings

Community structure is a prominent feature of the Montreal network, with the 3 major communities exhibiting substantially different epidemiological thresholds and dynamics. However, community structure alone is insufficient to produce multiwave epidemics because direct contacts between highly connected individuals in different communities can fuel rapid intercommunity transmission. It is the insularity of the most intraconnected community that makes multiple waves not just possible, but likely, when R_0_ resembles that estimated for the 1918 influenza pandemic. The Montreal network shows anecdotally that both strongly connected and insular communities can emerge naturally in human social systems and exist side-by-side. However, insularity is fragile, disappearing with minimal perturbation to the network. In contrast, the presence of strong bridges between communities is more robust and arises by random (or other) processes that link highly connected individuals to one another. Conversely, we expect that social differences, geographic heterogeneity in contact patterns (eg, city vs suburbs), and polarizing events would generate insularity.

The 3 large Montreal hotspot communities exhibit substantially different epidemiological characteristics, including epidemic thresholds and sensitivity to the reproduction number. For mildly transmissible diseases just above the epidemic threshold, some communities may be fully protected by the sparseness of their within-community network. Well above the epidemic threshold, the attack rate may vary considerably among communities, depending again on the structure of their within-community network.

Ball and Neal [40] introduced a theoretical framework addressing the epidemiological interplay between local and global connectivity, and showed that transient long-range connections can fuel epidemics even when local network structure is too sparse to sustain epidemics. Although their model considers transient global contacts rather than fixed community structure, a similar approach may ultimately provide a theoretical perspective on the epidemiological phenomena we observed in the Montreal network.

The insularity of the most intraconnected Montreal community is likely to produce a multiwave epidemic when an outbreak originates in that community and the R_0_ is moderate, close to that estimated for the 1918 influenza pandemic. For less contagious diseases, epidemics are unlikely to escape beyond this community; for highly contagious diseases, epidemics flow readily among communities. Our results thus support an alternative nontemporal explanation for multiwave epidemics—insular community structure—that arises naturally from social network structure fundamental to human interactions.

All historical influenza pandemics have produced multiple epidemic waves in many North American cities [1]. Specifically, Montreal experienced 2 distinct waves of influenza-related mortality during the 1957-1958 pandemic, which did not occur elsewhere in Quebec. McDonald [41] attributed this difference to population density. The 2009 pandemic also produced dual waves in Montreal [42]. Prior attempts to explain these patterns have largely overlooked population structure in favor of dynamic phenomena, such as pathogen evolution or host behavior [14,15,43,44]. This omission stems partly from the chronic lack of data and insight into the structure of urban contact networks. Although this remains a challenge, our study suggests that urban community structure may naturally produce multiwave pandemics, even without any temporal forcing.

### Limitations

The major limitation of our study lies in the inherent difficulty of capturing and characterizing human contact patterns at an individual level. The Montreal ÎSF network excludes many interactions important to disease transmission, such as those occurring in homes and schools. In addition, some of the colocation contacts in our dataset may not have been sufficient for disease transmission. For these 2 reasons, the ÎSF network does not fully reflect the human contact patterns responsible for disease spread; however, it reveals previously uncharacterized urban-scale community structure that may reflect fundamental geographic, economic, and cultural processes that shape physical contact networks.

### Conclusions

Anticipating the emergence of secondary epidemic waves is vital to epidemic and pandemic preparedness and response. For example, in the midst of an outbreak, it may allow public health officials to target interventions toward subpopulations still at risk for significant transmission. Identifying informative indicators of urban community structure, including the insularity estimates proposed in this study, will allow us to construct temporal epidemiological risk maps and target interventions (eg, vaccination) toward highly connected individuals reaching outside their own communities that can potentially serve as bridge superspreaders. In the Montreal network, communities had distinct geographic signatures, suggesting that investigations of community structure may also facilitate the effective placement of surveillance or intervention sites such as vaccination clinics.

## Appendix

Multimedia Appendix 1 Supplementary methods.

Multimedia Appendix 2 Supplementary figures.

## Abbreviations

CRAWDAD: Community Resource for Archiving Wireless Data at Dartmouth
ÎSF: Île Sans Fil
R_0_: basic reproductive number
