# Supplementary material for: Epidemic Wave Dynamics Attributable to Urban Community Structure: A Theoretical Characterization of Disease Transmission in a Large Network
Source: J Med Internet Res. 2015 Jul 8;17(7):e169. doi: 10.2196/jmir.3720 (PMC4526984; doi:10.2196/jmir.3720)
Supplement: Multimedia Appendix 1 [file jmir_v17i7e169_app1.pdf]

Multimedia Appendix 2: Supplementary Methods

Epidemic wave dynamics attributable to urban community structure

Anne G. Hoen, Thomas J. Hladish*, Rosalind M. Eggo, Michael Lenczner, John S. Brownstein, and Lauren Ancel Meyers

*Corresponding author. E-mail: tjhladish@ufl.edu

Detecting multiwave epidemics. To automatically identify epidemic curves exhibiting multiple waves, we define a new two-peak metric (*TP*), given by

$$TP(S) = \max_{1 \le i < j \le n} \left(\sqrt{(s_i - v_{i,j}) \times (s_j - v_{i,j})} \right) \tag{S1}$$

where *S* is the epidemic time series $(s_1, s_2, \dots s_n)$ and $v_{i,j} = \min_{i \le k \le j}(s_k)$ is the minimum value occurring in the time series between s_i and s_j. *TP* indicates the depth of the deepest valley in the epidemic time series (specifically, the geometric mean of the heights of on each side of the deepest valley).

Percolation-based approximations of R_0, epidemic size and community bridging. Given a semi-random network with degree distribution $\{p_k\}$ and a pathogen with per edge transmission probability *T*, the reproduction number is given by

$$R_0 = T\left(\frac{\langle k^2 \rangle - \langle k \rangle}{\langle k \rangle}\right) \tag{S2}$$

where $\langle k \rangle$ is the average degree and $\langle k^2 \rangle$ is the average squared degree [29]. To calculate global R_0, we consider the total degree of each node in the network; for community-specific R_0, we restrict to within-community degree. The expected size of an epidemic (proportion infected) is given by

$$S = 1 - \sum_{k=1}^{\infty} p_k \left(1 + (u-1)T\right)^k \tag{S3}$$

where $u = \dfrac{\sum_{k=1}^{\infty} k p_k \left(1+(u-1)T\right)^{k-1}}{\sum_{k=1}^{\infty} k p_k}$ is a self-referencing equation solvable by root finding methods [30]. For specific communities, we simply restrict to within-community degrees (e.g., denoted S_A and u_A for community *A*).

The probability that a node *a* in community *A* sparks an epidemic, once infected, is equal to one minus the probability that none of *a*'s contacts in *B* spark an epidemic (which occurs if transmission does not occur from *a* or if it does occur but fails to seed an epidemic in *B*), as given by

$$\varphi_a = 1 - \prod_{b \in \beta_a} \left((1-T) + T(1-\varepsilon_b) \right) = 1 - \prod_{b \in \beta_a} \left(1 - T\varepsilon_b\right) \tag{S4}$$

where β_a denotes the set of *a*'s contacts in *B*, $\varepsilon_b = 1 - \left(1 - T + Tu_B\right)^{\kappa_b}$ is the probability that *b* sparks an epidemic in *B* [29], and κ_b denotes the within-community degree of *b*. Early in an epidemic, the probability that a random transmission event will reach a given node is proportional to its degree [30]. Thus, the probability that an early transmission event within *A* will immediately seed an epidemic in *B* is given by

$$\phi_{A\to B} = \sum_{a\in A}\left(\frac{\kappa_a}{\sum_{a\in A}\kappa_a}\right)\varphi_a \, . \qquad \text{(S5)}$$

For an epidemic starting in *A* to cause a distinct secondary wave in *B*, it must eventually spark an epidemic in *B*, but not too early that it appears synchronous. This is approximately given by

$$\Gamma_{A\to B}(\gamma) = (1-\phi_{A\to B})^{\gamma} - \prod_{a\in A}(1-v_a\varphi_a) \qquad \text{(S6)}$$

where $v_a = 1-(1-T+Tu_A)^{\kappa_a}$ is the probability that *a* becomes infected during the epidemic within *A* [29]. The first term is the probability that the epidemic does not spread to community *B* before γ within-community *A* transmission events, and the second term is the probability that it never spreads to *B*. To calculate this probability for epidemics spreading from community III to I and from community I to II, we assumed $\gamma = 50$ and $\gamma = 200$, respectively, based on the range of lags observed between subsequent waves in our stochastic simulations (see Multimedia Appendix 1, Figure S1). Epidemics typically appeared synchronous if the source community seeded an epidemic in the recipient community within fewer transmission events.
