# Supplementary material for: Epidemic Wave Dynamics Attributable to Urban Community Structure: A Theoretical Characterization of Disease Transmission in a Large Network
Source: J Med Internet Res. 2015 Jul 8;17(7):e169. doi: 10.2196/jmir.3720 (PMC4526984; doi:10.2196/jmir.3720)

Multimedia Appendix 1: Supplementary Figures

Epidemic wave dynamics attributable to urban community structure

Anne G. Hoen, Thomas J. Hladish*, Rosalind M. Eggo, Michael Lenczner, John S. Brownstein, and Lauren Ancel Meyers

*Corresponding author. E-mail: tjhladish@ufl.edu

Figure S1: Example epidemic curves (R_0 = 2.4) classified according to the novel two-peak metric (*TP*), which characterizes the multimodality of an epidemic curve. Black lines show prevalence across communities, and bars show epidemic in each community, with blue, green and red representing communities I, II, and III, respectively. Epidemics with *TP* less than 1.5 were classified as single peaked, and those above 1.5 were classified as multi-wave. The statistic is conservative when determining multi-wave epidemics and is consistent across a range of R_0 values.

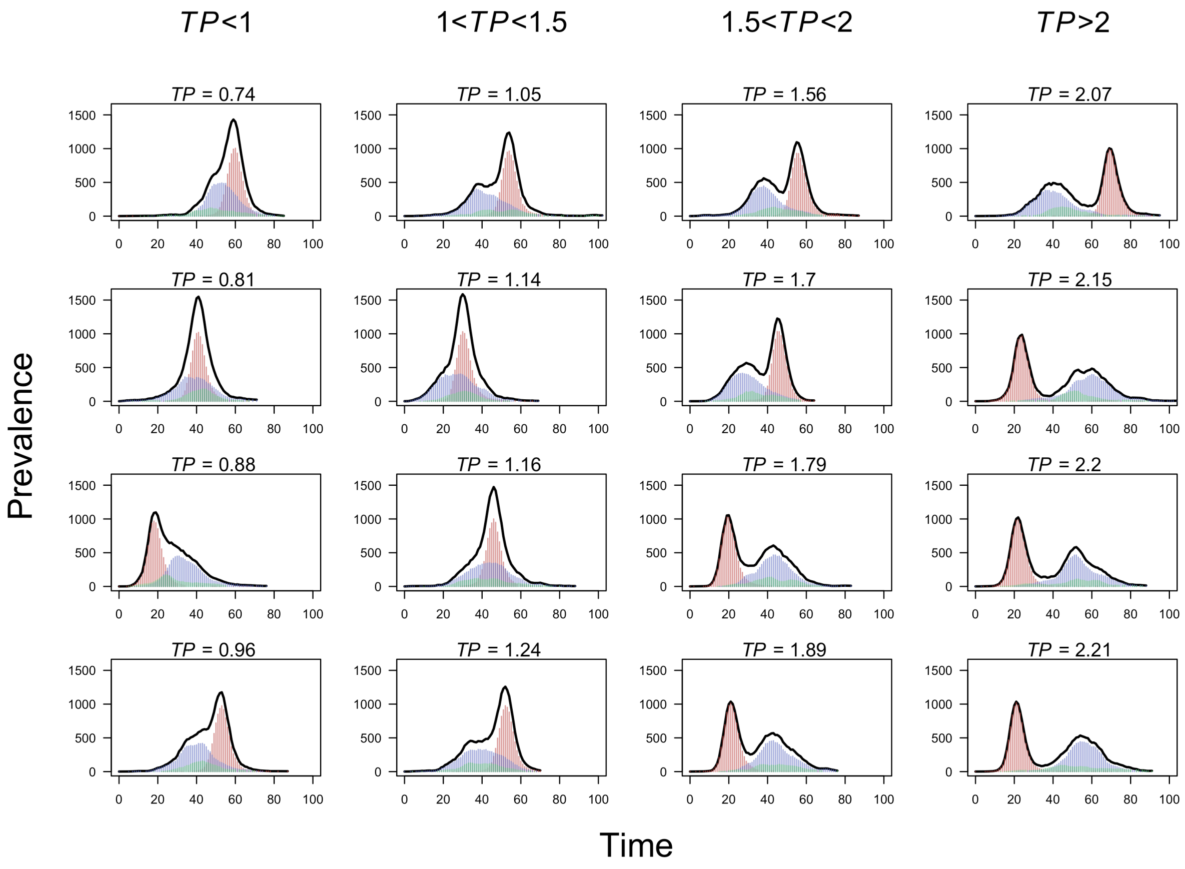

Figure S2: *TP* score distributions. Histograms show the distributions of *TP* scores for epidemics simulated at a range of R_0 values. As R_0 increases, the *TP* statistic for the subset of epidemics that are two-peaked also increases, but only to a point; it is constrained by the structure of the network as well as the chosen model parameters.

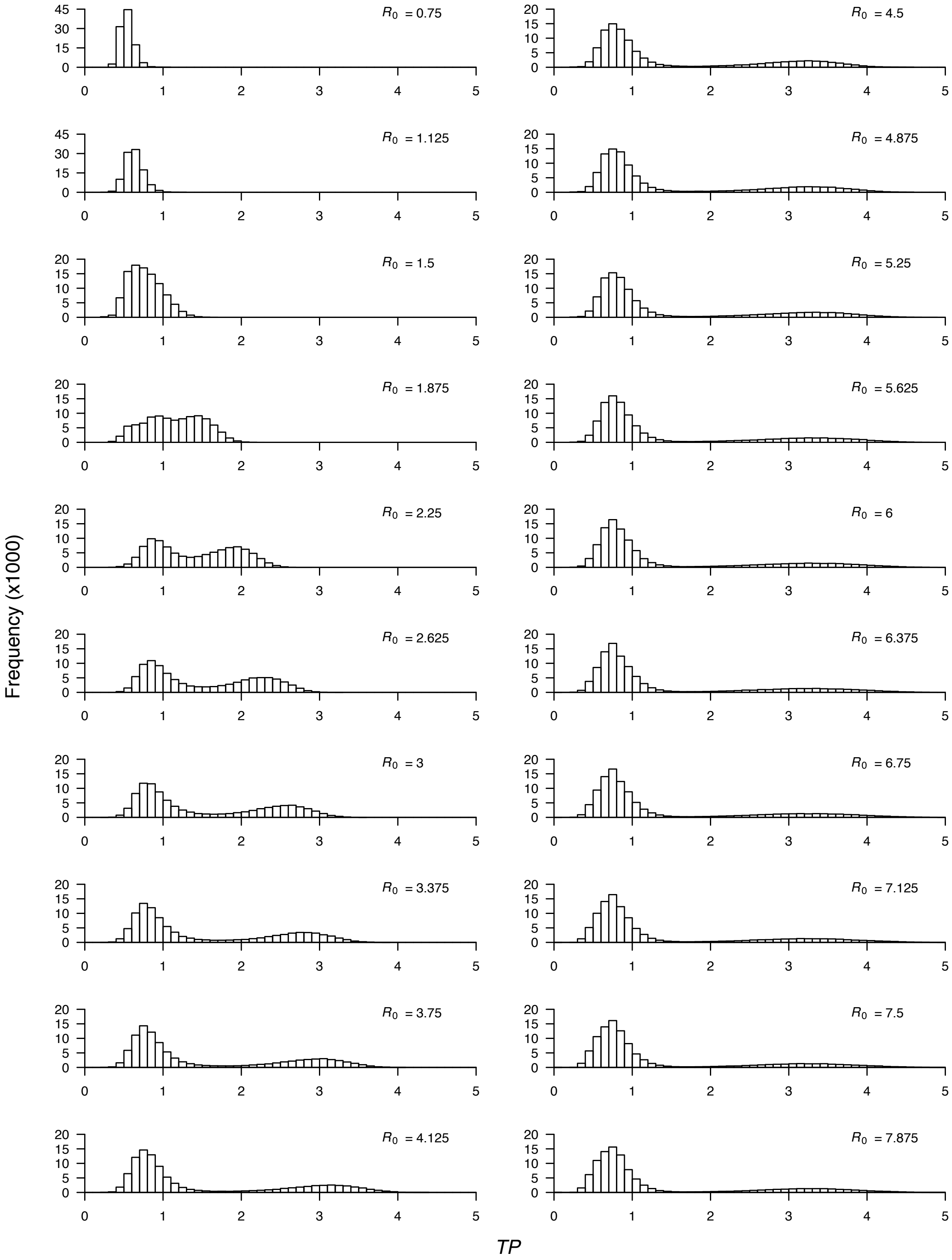

Figure S3: Histogram of community sizes (number of nodes). Although some communities contain more than 10,000 nodes, most have fewer that 10.

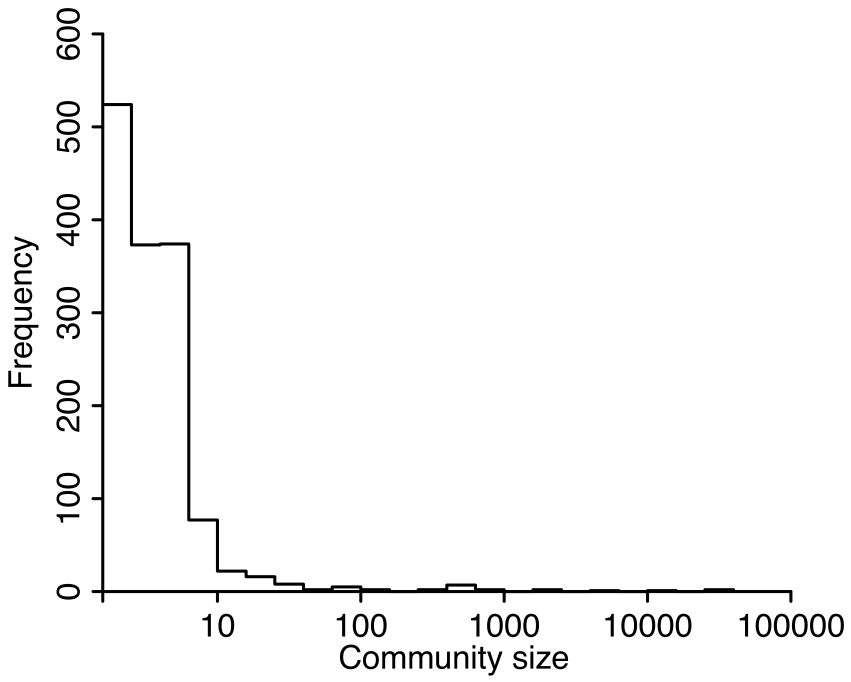

Figure S4: Effect of infectious period on multi-wave dynamics. For each infectious period, 10,000 epidemics were simulated and then classified as single-wave (left), multi-wave starting with a community III wave followed by a community I and II wave (middle), or multi-wave ending in a community III wave (right). Time series are plotted for a random subset of 1000 simulations per infectious period and were superimposed so that the peaks of the largest waves align. For each infectious period (1, 5, 10, or 50 time steps), the per-time-step transmission probability was adjusted to maintain a constant R_0 and expected final size. It should be noted that although the peak height appears to increase with the number of time steps in the infectious period, this is an aggregation artifact, and the final size of the epidemics did not change: all four infectious period scenarios had mean final sizes between 4426 and 4431 (n=10,000 for each).

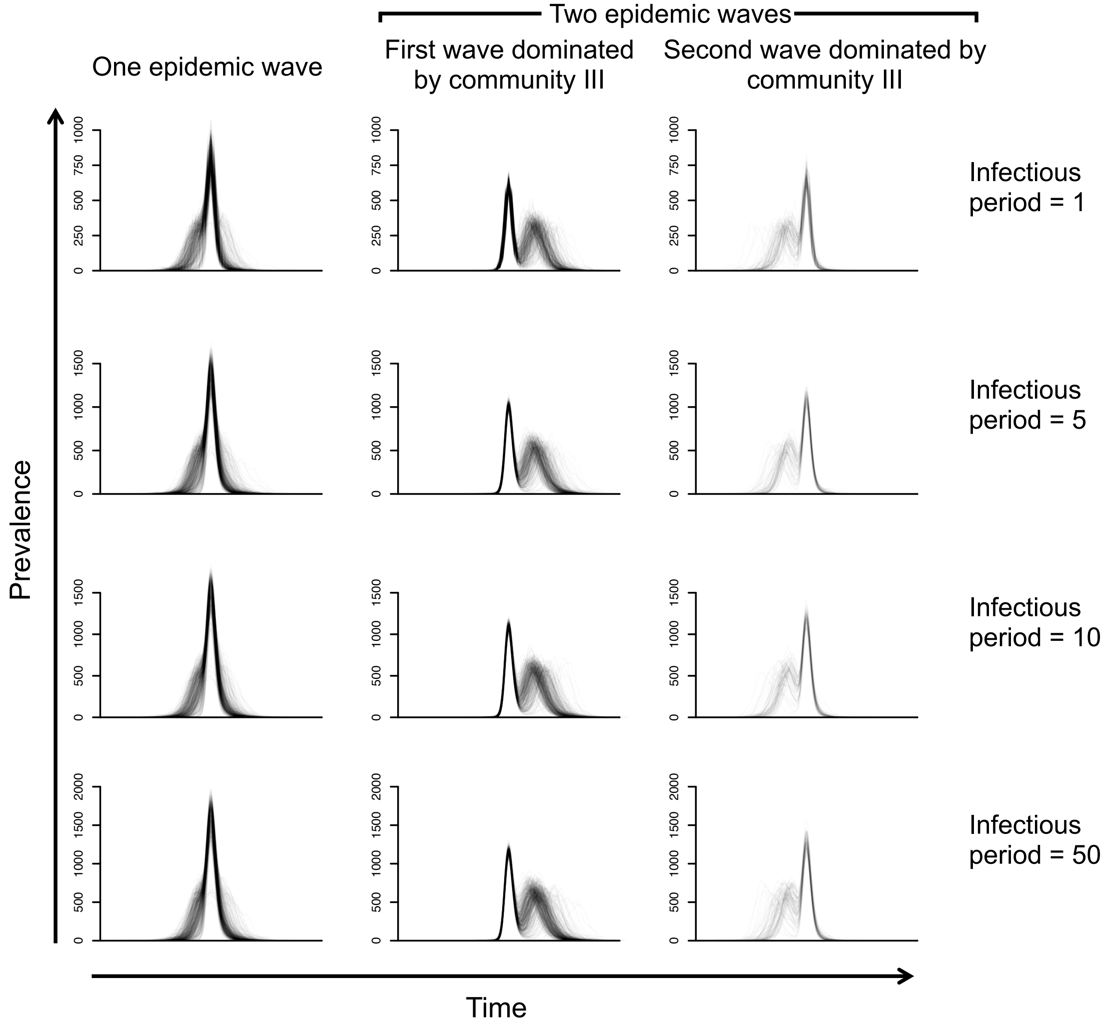

Figure S5: Changes in numbers of connections within individual modules and between module pairs with shuffling. The numbers of edges within and between communities change gradually and are robust to small amounts of shuffling.

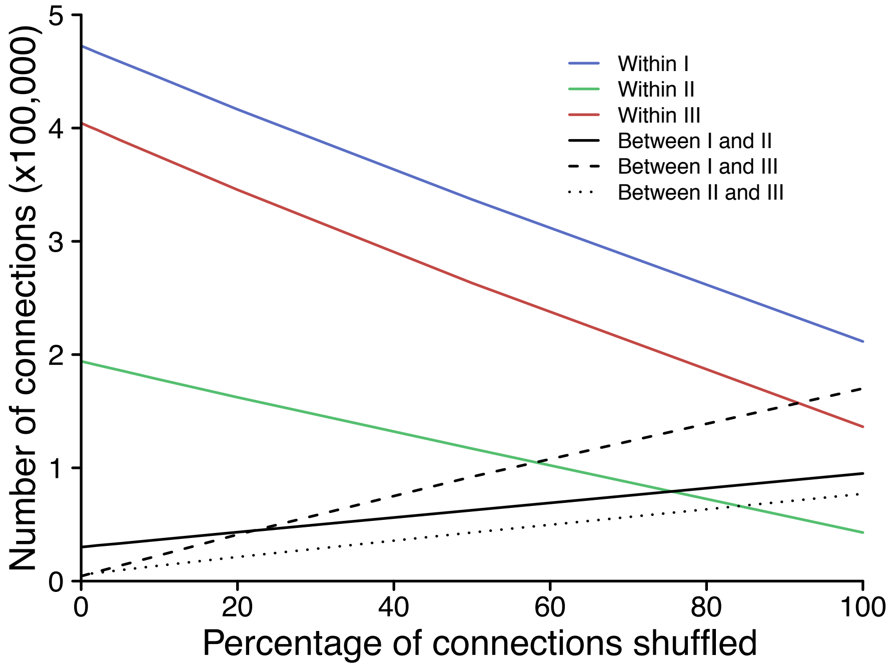

Figure S6: Network degree distributions. (**A**) Entire Montreal network and (**B**) individual communities (black lines: including connections to other communities; gray lines: excluding connections to other communities). The overall and within-community cumulative degree distribution functions (CDF) plotted on (**C**) linear-log and (**D**) log-log scales suggest that all four distributions are roughly power law with exponential cutoffs.

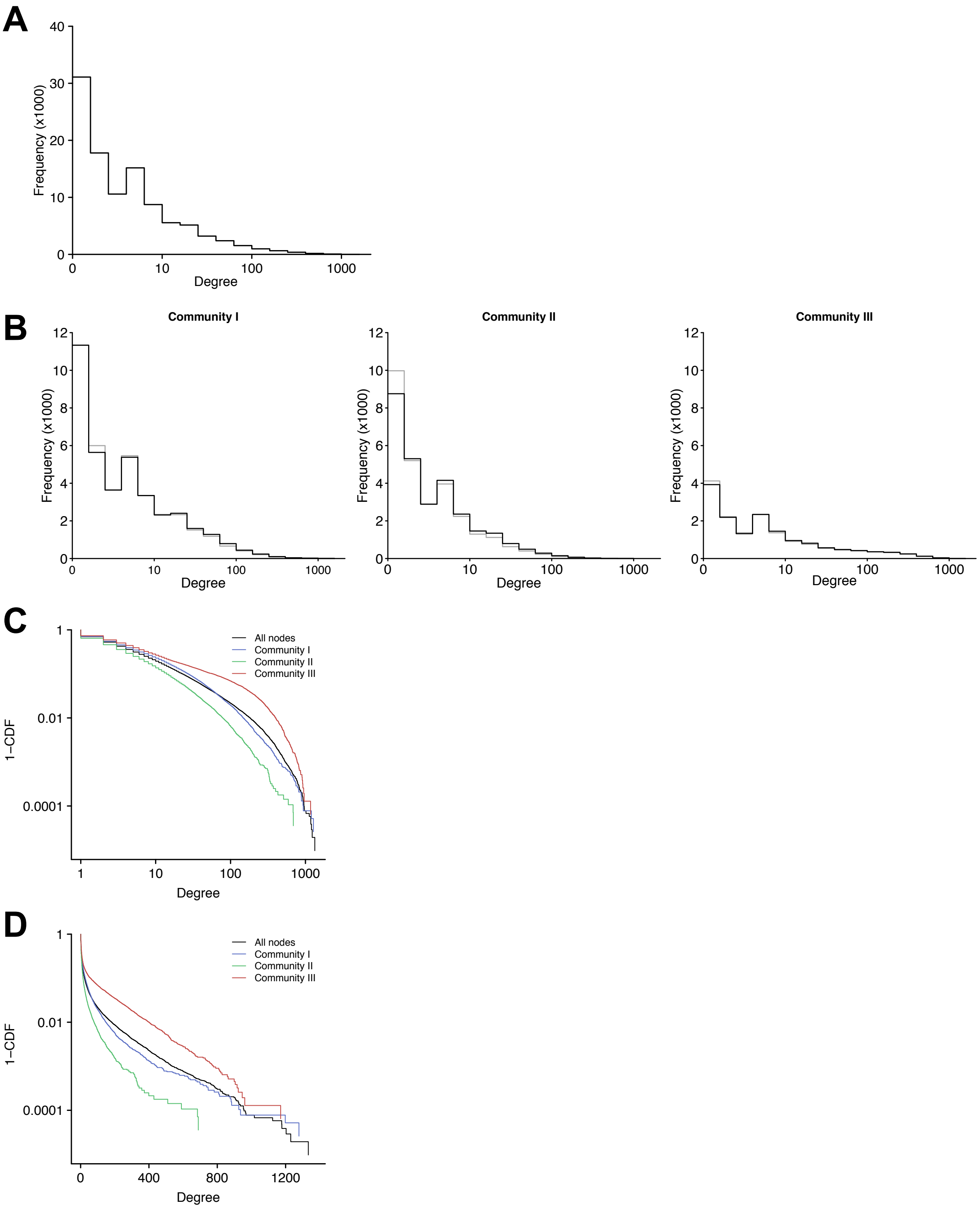

Supplement: Multimedia Appendix 2 [file jmir_v17i7e169_app2.pdf]
